# University of New York. Medical Department. Annual Announcement of Lectures. Session for 1846-7

**Published:** 1846-07

**Authors:** 


*University of New-York. Medical Department. Annual announcement of Lectures. Session for* 1846–7.—"As false as a Bulletin," became a proverb during the career of the great Napoleon, from the extravagant statements, and inflated style, which characterized the despatches announcing the triumphs of his military genius. It has sometimes occured to us that with the present captiousness prevailing in the ranks of the profession toward medical colleges there may be some danger of their circulars falling under a like implication. If the medical pupil were to be influenced solely by the protestations and inducements held out to him for his patronage in these annual announcements, we are quite certain that he would have some difficulty in making his selection. Each institution (if judged by the statements and panegyrics in which it is not unusual for them to indulge,) possesses such peculiar and superior advantages; each is so fortunate in the talents, zeal, and disinterestedness of its faculty, and in all the circumstances which can contribute to medical instruction, that we can easily imagine the medical student to be perplexed and embarrassed in making a choice among the many which manifest so much solicitude for his interests. In making these remarks we do not mean to be understood as asserting that all the medical schools of our country are in the habit of *puffing* their own merits and attractions. We are well aware that there are some, and perhaps many exceptions. But that the custom prevails, *usque ad nauseam*, in more than one institution, is too much the subject of comment with the profession to require proof. Nor do we intend by these remarks to insinuate that in these annual circulars false statements are given for facts, or promises made which are not fulfilled. We are not disposed to institute charges so grave as these. But we mean to imply that much is published which self-respect requires should not be spoken by the interested party, if said at all; and that the tone and style which characterise these documents are not in keeping with that dignity and propriety which should be expected from individuals of character and station in their associated, not less than in their individual capacity.

Our readers will perceive that we have prefixed to this article the title page of the circular of the medical department of the university of New-York, for 1846-7. We regret that this flourishing institution, in the metropolis of our state, furnishes so striking an illustration of the pertinency of our remarks. With its local recommendations, its great appliances and resources, and the reputation embodied in its faculty, it would surely seem that it might afford to dispense with that rhodomontade, which, although equally offensive, would be more excusable in some village school, which finds it necessary to supply in empty words what it lacks in substance. Yet there is more than one school, out of the metropolis, whose official announcements might be held up to the university of New-York, as worthy models for imitation. Not to confine ourselves to generalities, we quote the following portion of a paragraph as a specimen.

"A few years since, and New-York was known only as the *Commercial Emporium*; her fame in Medicine had not traveled beyond the confines of her own State; and her Medical classes were insignificant in numbers, composed mostly of students residing in the vicinity of the city. Now, her Medical reputation can compare with that of the oldest and most flourishing Institutions in the land. Her Alumni are scattered throughout the various

States; they have proved true to their trusts, and are worthy of the confidence reposed in them by their *Alma Mater*. Every year exhibits fresh evidence of their continued devotion to the interests of the University; and for their zeal and kindness the Faculty beg leave to return their acknowledgments."

Would any medical reader unacquainted with the fact, suspect, after perusing the foregoing, that New-York embraces another Medical College, having, of course, the same local advantages, which was in existence long before the organization of the Medical department of the University? We make no farther comment on this quotation, nor do we intend to criticise the circular in detail. We would simply farther say that it must be interesting information for the medical profession that the college building was purchased with the individual funds of the faculty, more particularly as it furnishes an additional interest in the fulfilment of their design, which is "*the building up a national school worthy of the country and the age*" The medical student, also, will be edified in reading that he need be at no trouble to provide for himself soap and towels in the dissecting room! But while on the subject we will allude to one thing which we had noted for comment some time ago. We observe on all the official publications which emanate from this school, an engraving of the University building, as a frontispiece. As it is given without any title, the inference naturally is, that this is the college edifice. We think there is a little deception here, which we do not say is intended, but which ought in propriety to be provided against. The building known as the Stuyvesant Institute is sufficiently commodious and convenient, but it is not the building printed on the title page. We have thus far referred, specifically and generally to offenses against courtesy and good taste, but we now quote from the same circular a paragraph which seems to us obnoxious to much graver criticism. It is as follows:

*Practical Anatomy.*—The facilities which the city of New-York presents for the study of Practical Anatomy are well known throughout the breadth of the land, Her immense population allows a large surplus of the *materiel*; and from this *surplus*, Schools of Medicine, in almost all parts of the country, have mainly drawn their supplies, thus instituting a traffic most dangerous to the interests of Practical Anatomy. The attention of the muncipal authorities has been drawn to the subject, and hereafter, it is understood, the *trade* will be at an end so far as the city of New-York is concerned. The arrangements for providing the University with the necessary *materiel* are perfect, and the students enjoy opportunities for the pursuit of practical Anatomy second only to those offered by the city of Paris. The *materiel* is not only abundant, but it is likewise extremely *cheap*, and is always furnished to the classes promptly and without fail, as often as it may be required.

This is certainly an extraordinary paragraph! The medical department of the University of New-York professes to have become alarmed for the interests of practical anatomy! And whence is the danger expected? From the application of the *surplus materiel* of the city of New-York to the study of anatomy in other schools! What a conjunction of cause and effect! what reasonable apprehensions! what excessive disinterestedness! But these fears have not expended themselves in silent inactivity. "*The attention of the municipal authorities has been drawn to the subject*:" Aye, drawn, too, as we are left to infer, by the medical department of the University of New-York; and "*the trade will be at an end.*" Consequently, in so far as the medical department of the University is concerned, "*the materiel is not only abundant, but it is likewise extremely cheap.*" In other words, in the very disinterested efforts "to make available the vast resources of the American Metropolis," the faculty have endeavored to create a monopoly in the trade of subjects for dissection. "*The arrangements for providing the University are perfect,*" but take care, agents for sister schools of medicine, the police are on the alert, and the medical department of the University of New-York are determined to have you caught and imprisoned if you make any attempt to avail yourselves of the "*large surplus*" with which the city of New-York abounds! This seems to us a fair interpretation of the ideas conveyed in this very singular announcement. We had written a page giving in plain terms our sense of its character, but we have drawn our pen across the lines, and will leave the reader to make his own comments.

In calling attention to this circular, we trust no one will do us the injustice to suppose that we entertain any feelings of hostility toward the institution in question. We can have no possible motives or occasion for them. We wish the institution success, and should rejoice to offer our humble congratulations upon its attaining that high position to which it professes to aspire. What has been said does not relate to the competency of its faculty, or to the advantages which it offers to the pupil: it may be in these respects what it claims to be. But whoever is responsible for the preparation of its circular for 1846–7 has not only, in our poor judgment, shown a want of delicacy and good feeling which is a fair subject of criticism, but has evinced a disregard of principles of medical ethics which are assuredly not less binding on a faculty of a medical school than on the individual practitioner.

